# Inhibition of HBV Expression in HBV Transgenic Mice Using AAV-Delivered CRISPR-SaCas9

**DOI:** 10.3389/fimmu.2018.02080

**Published:** 2018-09-11

**Authors:** Hao Li, Chunyu Sheng, Hongbo Liu, Shan Wang, Jiangyun Zhao, Lang Yang, Leili Jia, Peng Li, Ligui Wang, Jing Xie, Dongping Xu, Yansong Sun, Shaofu Qiu, Hongbin Song

**Affiliations:** ^1^Institute of Disease Control and Prevention, Academy of Military Medical Sciences, Beijing, China; ^2^State Key Laboratory of Pathogen and Biosecurity, Academy of Military Medical Sciences, Beijing, China; ^3^Research Centre for Liver Failure, Beijing 302nd Hospital, Beijing, China

**Keywords:** HBV, CRISPR-SaCas9, adeno-associated virus, HBsAg, gene therapy

## Abstract

The chronic production of hepatitis B viral (HBV) antigens could cause inflammation and necrosis, leading to elevation of liver enzymes from necrotic hepatocytes, hepatitis, cirrhosis, hepatocellular carcinoma, and liver failure. However, no current treatment is capable of significantly reducing HBsAg expression in patients. Our previous studies had confirmed the ability of CRISPR-Cas9 in disrupting HBV cccDNA. Here, to inhibit HBV expression efficiently in the mouse model of chronic HBV infection, the miniaturized CRISPR-SaCas9 system compatible with a HBV core region derived guide-RNA had been packaged in recombinant adeno-associated virus (AAV) type 8, which lowered the levels of serum HBsAg, HBeAg, and HBV DNA efficiently in HBV transgenic mice during 58 days continuous observation after vein injection. It further confirms the potential of the CRISPR-Cas9 technique for use in hepatitis B gene therapy.

## Introduction

Hepatitis B virus (HBV) infection is a serious worldwide public-health concern. Since 1982, hepatitis B vaccination has prevented the spread of hepatitis B to a substantial extent (1). However, the number of people with chronic HBV infection [hepatitis B surface antigen (HBsAg)-positive for at least 6 months] remains high. Globally, >688,000 people are estimated to die annually from the complications of chronic hepatitis B, including hepatocellular carcinoma and cirrhosis (2). The pathogenicity of HBV is mainly attributed to its cell-mediated immune response in liver cells; whereas HBsAg and HBeAg lead to hepatitis and an elevation of transaminase, HBcAg triggers the CD8 response of cytotoxic T lymphocytes and thus leads to the pathological damage of liver cells. Long-term chronic hepatitis leads to cirrhosis, liver cancer, and liver failure (3). In this way, the onset of hepatitis and cirrhosis can be effectively prevented by reducing serum HBsAg and HBeAg levels, as well as the HBcAg level in liver cells (3). However, currently available therapeutic drugs, such as interferon-α (IFN-α) and nucleoside analogs (NAs), cannot substantially reduce the serum HBsAg level in patients (4–6).

As a serial of studies, our previous studies had demonstrated that the CRISPR-Cas9 genome-editing technique could disrupt HBV cccDNA and eradicate integrated HBV DNA in cell line and hydrodynamic injection (HDI) mouse model (7, 8), Together with other previous studies, it had already been demonstrated that the CRISPR-Cas9 system is a potentially powerful tool capable of promoting a radical or “sterile” HBV cure as the proof of concept (7–12). However, up to now, there is no report that HBV expression could be efficiently decreased using CRISPR-Cas9 in mouse model of chronic HBV infection because an efficient and safe gene-transfer vector remains unavailable.

Recombinant adeno-associated virus (rAAV) is regarded as an optimal vector for gene therapy, and the recent discovery of the miniaturized CRISPR-SaCas9 (*Staphylococcus aureus* Cas9) system has offered a new tool for studies related to gene therapy performed using the CRISPR-Cas9 technique. Tristan Scott et al. have demonstrated that the AAV-delivered CRISPR-SaCas9 system could effectively disrupt HBV cccDNA and inhibits HBV replication in the HBV cell-line model (13). The most recently research showed that Liu et al. tried to inhibit HBV expression in the mouse model of chronic HBV infection using AAV delivered CRISPR-SaCas9, but the decrease of HBV antigens were not significantly different from the control (14).

Based on previous studies, using rAAV8::CRISPR-SaCas9, we have efficiently lowered serum HBsAg, HBeAg levels, HBV DNA and liver-cell HBcAg level in HBV transgenic mice without obviously off-target effect in this study, which demonstrate that HBV expression could been efficiently suppressed in this mouse model of chronic HBV infection using AAV delivered CRISPR-Cas9 and further confirms the potential of the CRISPR-Cas9 technique for use in hepatitis B gene therapy.

## Materials and methods

### Ethics statement

All procedures involving animals were approved by the Institutional Animal Care and Use Committee, Institute of Disease Control and Prevention, Academy of Military Medical Sciences (The permit number is IACUC of AMMS 10-2015-028). Animal studies were performed in strict accordance with the Regulations for the Administration of Affairs Concerning Experimental Animals approved by the State Council of People's Republic of China. All mice used for experiments were housed in cages in a controlled environment (22–25°C, 50% humidity, 12 h light/dark cycle) and were sacrificed under ether anesthesia. All the animal experiments in this study had been handled in Animal Biosafety Level 2 (ABSL-2) laboratory.

### Plasmid

The SaCas9 expression vector harboring inverted terminal repeat (ITR) sequences (pX601-AAV-CMV__NLS-SaCas9-NLS-3xHA-bGHpA:U6__BsaI-gRNA; plasmid #61591) was provided by the Feng Zhang laboratory, and the cDNA for transcribing single-guide RNA (sgRNA) was synthesized by Beijing Genomics Institute. The CRISPR-SaCas9 expression vector was constructed by ligating the double-stranded cDNA expressing the sgRNA into the *Bsa*I-digested px601-SaCas9 plasmid, as previously reported (15).

### Screening of candidate sgRNAs

293T cell line was purchased from the Cell Bank of Type Culture Collection of Chinese Academy of Sciences; the cell lines passed the testing for mycoplasma. Cells were cultured in DMEM/high-glucose medium containing 10% FBS, at 37°C and under 5% CO_2_ in an incubator. The target sites obtained from preliminary screening were ligated into the px601-SaCas9 expression vector, each of them were co-transfected with pGL3-HBV1.2 plasmid into 293T cells at same ratio and concentration respectively. Each group was assigned with 3 replicates, and the gRNA-empty group served as the positive-control group. HBsAg and HBeAg levels in the supernatants were determined using the ELISA accordance with the instructions (Beijing Wantai Biological Pharmacy Enterprise Co., Ltd.).

### T7EI assay

The genomic DNA of cells was extracted and purified using Cell and Tissue DNA Extraction Kit (BIOMED). The extracted DNA was PCR-amplified using the following primer pair: Sa4-T7EI F: TTGACTACTAGATCCCTGGATGCTG; Sa4-T7EI R: ACTCTTGGACTCCCAGCAATGTCAA. The PCR product was denatured at 95°C for 10 min, gradually cooled to room temperature, and then digested with T7EI enzyme (NEB) according to manufacturer instructions. The digested product was separated and visualized in 2% agarose gels and the mutation rate was calculated based on the grayscale intensity of bands as follows: % gene modification = 100 × (1 – (1 – fraction cleaved)1/2).

### Construction of rAAV8 CRISPR-SaCas9

The SaCas9 expression vector harboring inverted terminal repeat (ITR) sequences (pX601-AAV-CMV__NLS-SaCas9-NLS-3xHA-bGHpA:U6__BsaI-gRNA; plasmid #61591) was provided by the Feng Zhang laboratory, and the cDNA for transcribing single-guide RNA (sgRNA) was synthesized by Beijing Genomics Institute. The CRISPR-SaCas9 expression vector was constructed by ligating the double-stranded cDNA expressing the sgRNA into the *Bsa*I-digested px601-SaCas9 plasmid, as previously reported (15).

The rAAV8::CRISPR-SaCas9 virus was packaged using the triple plasmid transfection method. The CRISPR-SaCas9 expression vector carrying ITR sequences at both ends was cotransfected with pAAV-RC packaging plasmid and pHelper helper plasmid into HEK293 cells. They were then cultured for 48 h before harvesting and purifying the virus by using cesium chloride in Beijing Five Plus molecular Medicine Institute. The virus was obtained at a titer of 1 × 10^12^ v.g.

### HBV transgenic mice

HBV transgenic mice were purchased from Beijing Vitalstar Biotechnology Co., Ltd. The HBV transgenic mouse model was constructed through pronuclear microinjection of the linearized HBV genome (GenBank ID: AF305422.1, subtype adw2, genotype A) at 1.28-fold length into the fertilized egg of a C57BL/6 mouse, and has stably transmitted HBV to the fifth generation. All mice were housed under controlled ambient illumination on a 12/12-h light/dark cycle. Mice were divided randomly in two groups and each group had 7 biological replicates.

### *In vivo* administration of rAAV8::CRISPR-SaCas9

Mice were randomly grouped into the experimental and control groups for intravenous tail injection with the virus by using BD insulin syringes as follows: Each group included 7 HBV transgenic mice. All mice were monitored continually for 6 h after injection. Blood was drawn from the ophthalmic vein of mice on the 3rd day after the first injection and on the 1st, 3rd, 5th, 7th, 10th, 14th, 18th, 24th, 38th, and 53th days after the second injection. Three randomly chosen mice from both the experimental and control groups were sacrificed on the 39th day after the second injection and were dissected to collect their heart, liver, spleen, lungs, and kidneys for the preparation of tissue sections and DNA extraction.

### Detection of HBsAg/HBeAg and HBV DNA

HBsAg and HBeAg levels in mouse serum were quantified according to the instructions provided with the Abbott Hepatitis B Virus Antigen Assay Kit (chemiluminescent microparticle immunoassay). The HBV DNA level in mouse serum was determined using a Hepatitis B virus (HBV) nucleic acid quantitative detection kit (Beijing SinoMDgene Technology Co., Ltd.), on a BioRad fluorescence-based quantitative PCR system (14.0), with the following reaction steps: 45 cycles of 37°C for 2 min, 95°C for 3 min, 95°C for 10 s, and 60°C for 35 s, followed by 72°C for 5 min and 25°C for 10 s.

### Immunofluorescence analysis

The dewaxed and rehydrated tissue sections were incubated for 30 min in a solution containing bovine serum albumin, and then incubated overnight at 4°C with the primary antibody diluted in phosphate-buffered saline (PBS, pH 7.4). On the next day, the slides bearing the sections were rinsed (3×, 5 min each) in PBS with agitation on a decolorization shaker. After the tissue sections were dried, a mouse secondary antibody was added dropwise and the slides were incubated in the dark at room temperature for 50 min. For single fluorescence labeling, tissue sections were incubated with the mouse secondary antibody, whereas for double labeling, tissue sections were incubated with a rabbit secondary antibody, and here HBcAg was labeled with FITC and hemagglutinin (HA) was labeled with Cy3. The tissue sections were repeatedly rinsed with PBS and then the DAPI staining solution was added dropwise, and this was followed by incubation in the dark at room temperature for 10 min. Lastly, the tissue sections were rinsed again with PBS and dried briefly, and then mounted with antifade mounting medium. The mounted tissue sections were examined under a Nikon inverted fluorescence microscope, and images were collected at UV-excitation wavelength of 330–380 nm and emission wavelength of 420 nm, and at FITC (green)-excitation wavelength of 465–495 nm and emission wavelength of 515–555 nm.

### HE staining and microscopy

The nuclei and the cytoplasm in the dewaxed tissue sections were stained with HE dye, after which the stained sections were dehydrated by immersing twice (5 min each) in 95% alcohol, anhydrous ethanol, and xylene, and then further dehydrating in xylene. The dehydrated and stained tissue sections were mounted in neutral gum and then examined and imaged under the Nikon inverted fluorescence microscope; the nuclei were stained blue, whereas the cytoplasm was stained red or green.

### DNA isolation and deep sequencing

Fresh tissues obtained from the dissected mice were rinsed repeatedly with PBS, minced, and ground in a tissue grinder to prepare homogenates for DNA extraction. Genomic DNA was extracted from mouse cells and tissues according to instructions provided with the Tissue and Cells DNA Extraction Kit (BIOMED). Subsequently, PCR was performed using Premix ExTaq (Takara Bio, Inc.). The purity and concentration of the PCR products purified using the Promega Gel Extraction Kit were measured using a Nanodrop instrument (Thermo Fisher Scientific, USA). Paired-end sequencing was performed on the multiplex libraries on an Illumina-Miseq PE300 platform with a minimum sequencing depth of 7000×. Moreover, the reads were demultiplexed and the read orientations were corrected according to the barcode and sequencing priming sites at both ends of reads. The barcode sequences allowed no more than one mismatched base.

### Statistical analysis

The quantitative data of HBV DNA, HBsAg, and HBeAg In animal experiments were shown as the mean ± standard deviation of 7 replicates before the 38th day and 4 replicates at the 53th day after the second injection. The Immunofluorescence analysis of HBcAg and the HBV DNA mutation rate analyzed using deep sequencing were shown in 3 replicates. Student's *t*-test was processed using the SAS software suite. *P*-values < 0.05 or 0.01 were considered statistically significant.

## Results

### Screening for efficient HBV target sites suitable for the CRISPR-SaCas9 system

**Figure 1:**
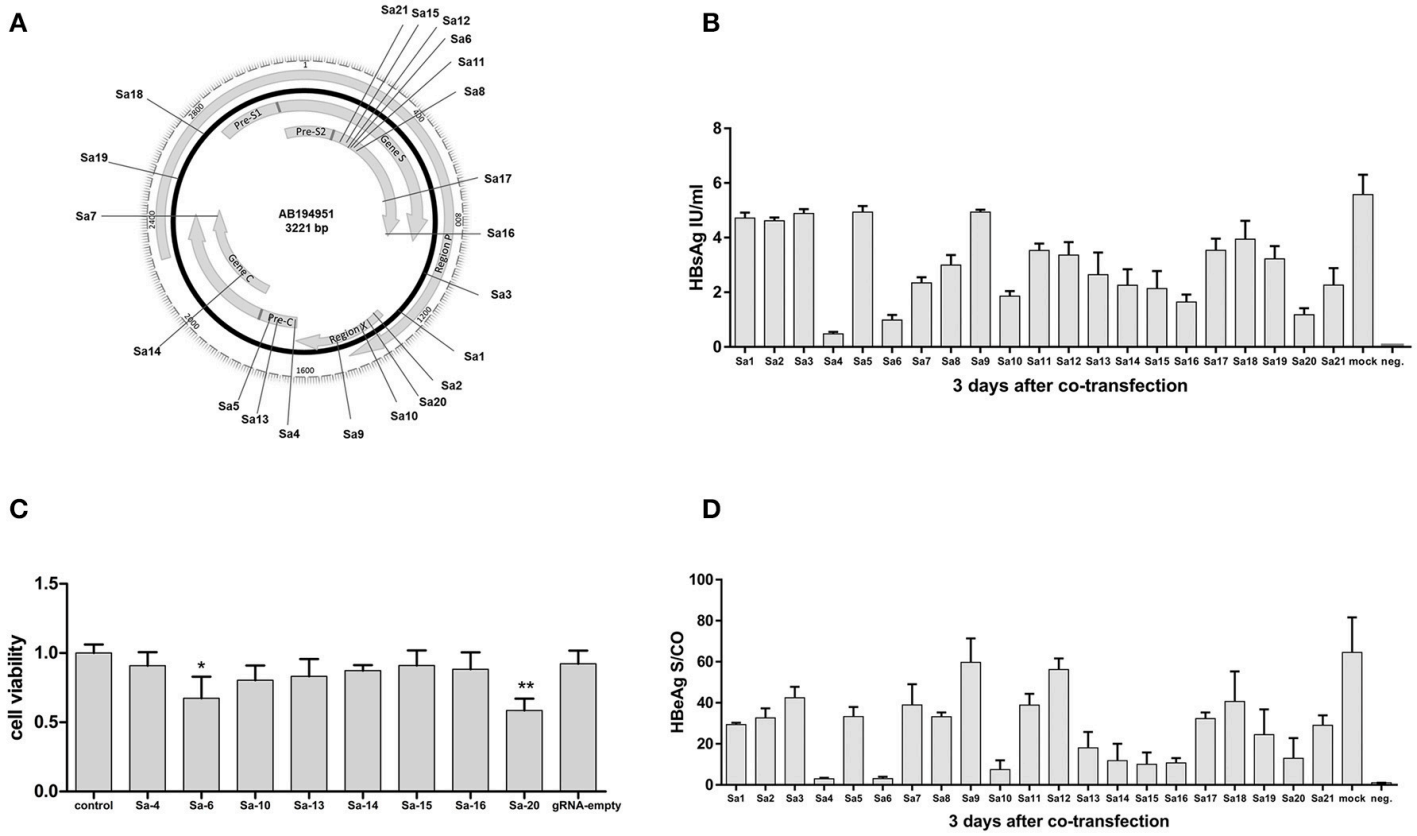
Identification of an effective HBV-specific CRISPR/SaCas9 system. **(A)** Illustration of gRNA-targeted sequences located in the HBV genome. **(B)** Results of HBsAg measurement in cell-culture supernatants on the 3rd day after co-transfection with CRISPR-SaCas9 and pGL3-HBV1.2 expression vector in 293T cells. Error bars, SD; *n* = 3. **(C)** CCK-8 assay performed at the indicated time points after transfection (absorbance at 450 nm, A450). **(D)** Level of HBeAg in cell-culture supernatants on the 3rd day after co-transfection with CRISPR-SaCas9 and pGL3-HBV1.2 in 293T cells. Error bars, SD; *n* = 3.

Unlike the CRISPR-SpCas9 target site that requires the Protospacer Adjacent Motif (PAM) “NGG,” the CRISPR-SaCas9 target site requires the PAM “NNGRRT.” To obtain efficient SaCas9 target sites that are applicable to different HBV genotypes, we compared the genomic DNA sequences of 26 HBV genotypes (A-G) originating from distinct regions and identified 21 target sites in highly conserved regions on different genes of the HBV genome (Figure 1A, Supplementary Tables 1, 2). Comparison with the guide-RNA (gRNA)-empty-transfected control group revealed that 5/21 CRISPR-Cas9 systems (Sa4, Sa6, Sa10, Sa16, and Sa20) reduced the average HBsAg level in the supernatant by more than two-thirds (Figure 1B), and 8/21 CRISPR-Cas9 systems (Sa4, Sa6, Sa10, Sa13, Sa14, Sa15, Sa16, and Sa20) reduced the average HBeAg level by more than two-thirds (Figure 1D). Furthermore, we examined cell viability by using the Cell Counting Kit-8 (CCK-8) method to ascertain whether the inhibition of HBV by the aforementioned 8 CRISPR-SaCas9 systems was due to the suppression of cell viability. The results showed that relative to the viability in the control group (lacking gRNA target sites), cell viability was not affected in a statistically significant manner by any of the target sites, except Sa6 and Sa20 (Figure 1C). Among the 21 target sites examined, the Sa4 site showed highest inhibitory efficiency against HBV without affecting cell viability. Accordingly, we packaged the CRISPR-SaCas9 system carrying the Sa4 target site which showed highest inhibitory efficiency against HBV without affecting cell viability into rAAV for further studies.

### rAAV8::Sa4 efficiently suppressed the levels of HBV markers in the serum of HBV transgenic mice

The results of DNA sequencing and serological testing confirmed that all HBV transgenic mice used in this study contained the HBV genome at 1.28-fold length, and that the serum levels of HBsAg, HBeAg, and HBV DNA were 4,000–6,000 IU/mL, 800–1,000 S/CO (Sample/Cut Off), and >10^8^ v.g./mL, respectively. According to previous genome-editing studies, rAAV was administered at high dosage multiplicity of infection/mouse (16). In this study, after two successive viral injections at a dose of 1 × 10^11^ v.g./mouse twice with a 5-day interval, we found the serum level of the HBV markers in the mice of the experimental group decreased continuously over time (the level of each marker began to decrease at different time points); by contrast, the serum level of the HBV markers in the control-group mice increased over time.

**Figure 2:**
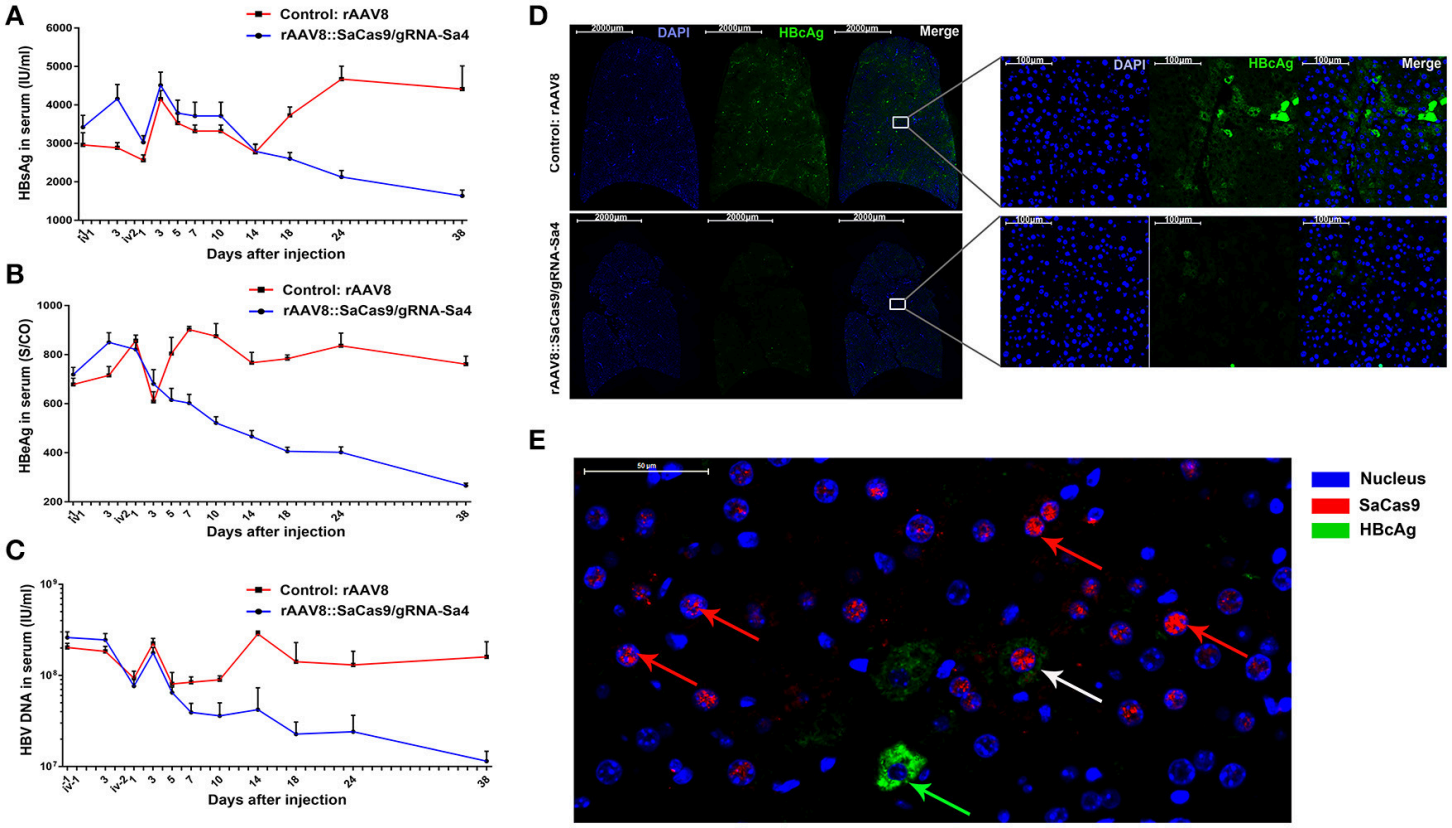
Inhibition of both HBV antigen expression and HBV replication by rAAV8::CRISPR-SaCas9 in HBV transgenic (HBV-Tg) mice. **(A)** Comparison of serum HBsAg levels measured in rAAV8-empty- and rAAV8::Sa4-treated groups at the indicated times after injection. **(B,C)** Comparison of serum HBeAg and HBV DNA measured in the aforementioned two groups at the indicated times after injection. **(D)** The results of immunocytochemistry/immunofluorescence (ICC/IF) analyses, performed with FITC-conjugated anti-HBcAg antibody. **(E)** Immunofluorescence labeling with anti-HA and anti-HBcAg antibodies showing the levels of SaCas9-HA (Cy3; red) and HBcAg (FITC; green) in the liver of HBV-Tg mice at Day 38 after injection; nuclei are stained blue with DAPI. In most of the liver cells of HBV-Tg mice transfected with rAAV8::CRISPR-SaCas9, HBcAg was not detectable (red arrows) or was less detectable (white arrow). While, an HbcAg-expressing liver cell (green arrows) has been found that didn't transfected with SaCas9.

In the following description of the results, we use “ivM-dN” to denote “the Nth day after the Mth injection.” Before iv2-d14, mice from both groups showed a similar trend with respect to changes in the serum HBsAg level (Figure 2A). However, the serum HBsAg level in mice of the experimental group decreased continuously starting from iv2-d3, and at iv2-d14, reached a level lower than that before injection (*P* < 0.05). Moreover, at iv2-d38, the serum HBsAg level in the experimental-group mice was 52.22 ± 9.79% and 62.96 ±7.59% lower than the level before injection and the level of the control group, respectively. In the control-group mice, the serum HBsAg level decreased after iv2-d3 but increased continuously after iv2-d14 and thereafter remained higher than the level in the experimental-group mice (*P* < 0.01), and at iv2-d38, the level was 32.91 ± 12.35% higher than the level before injection (Figure 2A). In the case of serum HBeAg (Figure 2B), the level measured in the mice of the experimental group at iv1-d3 was higher than the level before injection (*P* < 0.05), but it decreased continuously thereafter until iv2-d38, when the level was 63.09 ± 3.26% and 65.18 ± 3.08% lower than the level before injection and the level of the control group, respectively. Conversely, the serum HBeAg level in the control-group mice showed an overall increasing trend initially and remained at a steady level thereafter, with the level measured at each time point being higher than that in the experimental group from iv2-d7 onward (*P* < 0.01) (Figure 2B). Moreover, the differences between the two groups became larger over time. Lastly, the serum HBV DNA levels (Figure 2C) measured in the mice of the experimental and control groups were not significantly different at iv1-d3 as compared with the level before injection (*P* > 0.05). However, at iv2-d1, the serum HBV DNA levels in the experimental- and control-group mice were 70.62 ± 5.08% and 54.05 ± 8.93% lower than the levels before injection, respectively. At iv2-d3, the serum HBV DNA level in the experimental-group mice had returned to the level before injection (*P* > 0.05), but then decreased continuously until iv2-d38, when the level was 94.42 ± 1.24% and 92.82 ± 3.67% lower than the level before injection and the level of the control group, respectively (Figure 2C). By contrast, the serum HBV DNA level in the control-group mice remained steady after returning to the level before injection at iv2-d3 (*P* < 0.05), and from iv2-d7 onward, the level remained higher than that of the experimental group (*P* < 0.01).

To continue evaluating the inhibitory effects of AAV-delivered CRISPR-SaCas9, HBsAg, HBeAg, and HBV DNA level had been monitoring in the remaining 4 mice (3 mice were sacrificed for deep sequencing at iv2-d39) in each group at iv2-d53, the serum HBsAg, HBeAg, and HBV DNA level in the experimental-group mice was 35.59 ± 17.41%, 62.60 ± 2.50%, and 90.71 ± 5.37% lower than the level in control group respectively (Supplementary Figure 1). It indicates that the injection of rAAV8::Sa4 at 2 × 10^11^ v.g./mouse could continuously suppress the serum levels of HBsAg, HBeAg, and HBV DNA in HBV transgenic mice in the following 53 days after injection.

### Liver histopathology and immunofluorescence detection of HBcAg in HBV transgenic mice

HBcAg was distributed in the cytoplasm of HBV transgenic mice; thus, we dissected 3 mice randomly selected from each of the experimental and control groups at iv2-d38, and performed immunofluorescence analysis to determine the HBcAg level in their liver cells. The obtained panoramic images (Figure 2D) showed that the fluorescence intensity of HBcAg staining in liver tissues from the experimental-group mice was 76.88 ± 18.39% lower than that measured in the control group, which means that rAAV8::Sa4 could not only lowered the serum levels of HBV markers in mice, but also efficiently inhibited HBcAg expression in the liver cells of mice (Figure 2D). To evaluate the different tissue tropisms of rAAV type VIII (rAAV8), we also performed immunofluorescence analysis to determine the SaCas9 level in liver, kidney, heart, spleen and lung of HBV-tg mice. The results showed that rAAV8 could specifically infect liver tissue and relatively lower infectivity toward other tissues and organs (Supplementary Figure 2). Furthermore, the results of histopathological analysis, performed using hematoxylin-eosin (HE) staining showed that no pathological changes were detected in the liver, heart, spleen, lungs, and kidneys from the mice of both groups (Supplementary Figures 3–7).

### Detection of CRISPR-SaCas9 and HBV DNA mutations in the liver tissue of HBV transgenic mice

**Figure 3:**
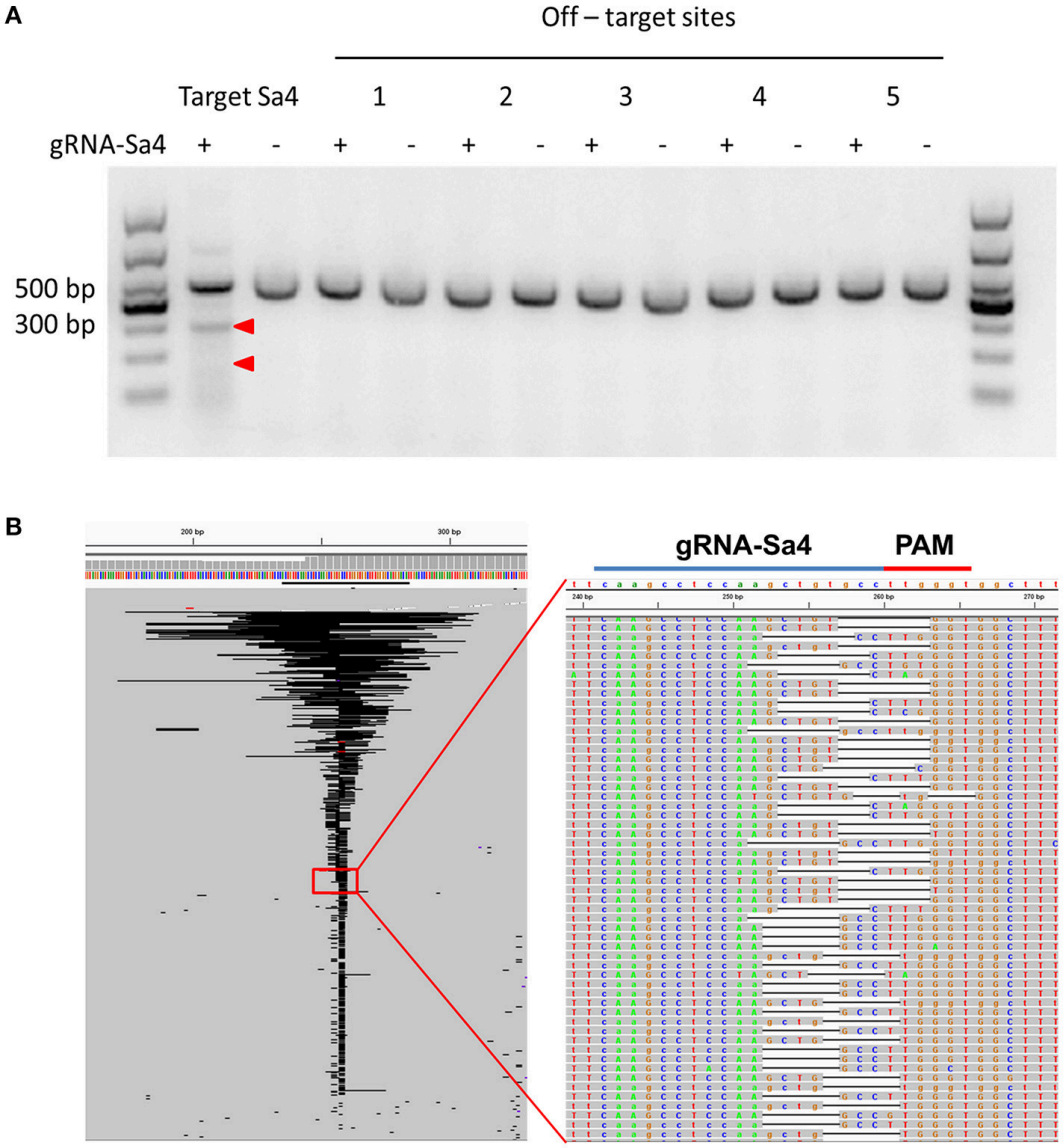
rAAV8-mediated CRISPR-SaCas9 introduction disrupted HBV DNA in the liver of HBV-Tg mice. **(A)** Assessment of off-target cutting by gRNA-Sa4. The results of the surveyor assay in liver cells treated with gRNA-empty (–) and gRNA-Sa4 (+) are shown; gRNA-Sa4 and gRNA-Sa4 off-target sites 1, 2, 3, 4, and 5 were PCR-amplified. T7EI nuclease-cleaved gRNA PCR products are indicated by Red arrow heads. **(B)** Representative views of target S4 region deletion in gRNA-S4-treated mice, generated using Integrative Genomics Viewer. Black bars: deletions; gray bars: sequencing reads.

To examine the rAAV8::CRISPR-Cas9 system in the liver tissue of HBV transgenic mice, we performed immunofluorescence analysis on liver tissue sections from the experimental-group mice and the results showed that SaCas9 was expressed in 57.63 ± 6.47% of the cells and was mainly localized within the nucleus at 38 days after the viral injection. And HBcAg was not detected in the majority of cells expressing SaCas9 (Figure 2E). Next, the HBV DNA-cleavage activity and predicted off-target (Supplementary Tables 3, 4) effect of gRNA-Sa4 was examined using T7EI analysis (Figure 3A). The results of T7EI digestion revealed the occurrence of indel mutations within the corresponding regions of the HBV genome in the liver tissues of the experimental-group mice; the frequency of the mutations caused by gRNA-Sa4 in the corresponding regions of the HBV genome was 41.05%. And we did not detect any indels caused by CRISPR-SaCas9 cleavage in the potential off-target sites. Besides, the deep-sequencing results showed that multiple indels of distinct lengths were present within the gRNA-Sa4 target region of the HBV genome in the liver tissue of the experimental-group mice (Figure 3B); the insertions and deletions ranged in length from 1 to 86 bp and 1 to 146 bp, respectively. It suggest that intravenous tail injection of the rAAV8::CRISPR-Cas9 system leads to high-efficiency cleavage of HBV DNA in the liver tissue of mice, and thereby disrupts the replicative template of HBV.

## Discussion

CRISPR-SaCas9 systems carrying gRNA-Sa4 efficiently inhibited the expression of both HBeAg and HBsAg from HBV DNA in mice, it possibly due to the circular replication intermediates of HBV DNA rapid cleavage efficiency resulting in a high percentage of linear DNAs that is not repaired but rather destroyed (17). Besides, as gRNA-Sa4-targeted region is PreC region which is also a coding region for virus enhancers, X protein, and core protein, which all play crucial roles in HBV replication and integration(18), the disruption of this region might markedly affect HBV replication and expression. Besides, a comparison of the genome sequences of 26 distinct HBV genotypes (A-G) from different sources revealed that only 3 HBV genotypes contained 1–2 point mutations within the Sa4 region and these mutations occurred at least 7 nucleotides from the PAM sequence. The results in this study indicates that the gRNA-Sa4 target site is suitable for the cleavage of the DNA of multiple HBV genotypes and is an optimal target site for the miniaturized CRISPR-SaCas9 system used for inhibiting HBV replication.

Distinct AAV serotypes exhibit varying tissue tropisms (19). For instance, AAV1 is particularly efficient to drive gene expression in the brain (20, 21), whereas AAV8 appears well suited for the transduction of liver (22, 23). AAV9 has the general ability to transduce all major tissues, including muscle (24), retina (25), heart (26), and lung (20) in mice. Thus, in this study, to minimize the effect of the CRISPR-SaCas9 system on organs other than the liver in mice, the CRISPR-SaCas9 system was packaged into rAAV8. As expected, we could hardly detected SaCas9 expression in kidney, heart, spleen and lung of HBV-tg mice using immunofluorescence analysis, while the level of SaCas9 in liver was much high. Additionally, the results also show that no pathological changes were detected in those tissues. Those benefits are important attributes that make rAAV8 delivered CRISPR-SaCas9 safe and efficient therapeutic options.

In previous genome-editing studies, rAAV was administered at a high dose in animals by using AAV::CRISPR-SaCas9: Ran et al. administered rAAV9 at 2 × 10^11^ v.g./mouse in their investigation of the use of rAAV9::SaCas9-gRNA to target the mouse PCSK9 gene(15); and Kaminski et al. used rAAV9::SaCas9-gRNA to inhibit the expression of HIV in HIV Tg26 transgenic mice by injecting the virus at 10^12^ multiplicity of infection/mouse, twice with a 5-day interval (16). Here, we also injected with rAAV at low doses (5 × 10^10^ v.g./mouse, each time). In pre-experiment, mice were injected with rAAV::CRISPR-SaCas9 at 5 × 10^10^ v.g./mouse and HBV DNA level did not differ between the experimental and control groups, which indicated that injection at 5 × 10^10^ v.g./mouse was insufficient for efficiently inhibiting HBV replication in mice. Thus, we subsequently injected the virus twice at 1 × 10^11^ v.g./mouse each time, with a 5-day interval, which resulted in a drastic reduction in the serum HBV DNA, HBsAg, and HBeAg levels in the mice of the experimental group, and this reduction continued until the 53th day after injection. It indicates that the administration dose of rAAV8 affects its inhibitory efficiency against HBV in mice, and that comparatively higher doses and repeated injections might be required to further reduce the HBV level in mice.

Off-target effect is a major risk of CRISPR/Cas9 technology. Even though CRISPR/Cas9 predominantly recognizes the intended target sites, a series of high-throughput genome-wide methods (27), Cas9 toxicity screens (28), and SITE-seq biochemical methods (29) have revealed evidence of off-target effects due to target mismatch tolerance of CRISPR/Cas9. Those unexpected off-target could cause structural chromosomal rearrangements of host cell, which may result in oncogenes activation or cause genome instability (30). In this study, though we analyzed the off-target effect of gRNA-Sa4 in mouse liver, the detection systems is not optimized for clinical applications. Fortunately, as the technology development, there are many ways to minimize CRISPR/Cas9 off-target effects in the human genome. For example, CIRCLE-seq approach could be used to identify genome-wide off-target mutations of CRISPR/Cas9 that are associated with cell type-specific single-nucleotide polymorphisms to provide personalized specificity profiles (31). Besides, a recently developed RNA-targeting Cas9 (RCas9) system could avoid permanent off-target genetic lesions in DNA-mediated CRISPR-based therapeutics (32). Beyond that, risks of germline transmission are also critical challenges to the safety of this strategy. Hence, the anti-HBV strategy in this study is being assayed for research purposes only and the risks of this new technology must to be fully resolved before its clinical application.

Based on the findings of this study, we firstly demonstrated that the AAV delivered CRISPR-SaCas9 could efficiently inhibit serum HBsAg and HBeAg in HBV transgenic mice as a proof of concept. However, this study still has some limitations such as lacking of long-term inhibitory effects evaluation and comprehensive analysis of host immune response. Although, the HBV transgenic mouse model offers a direct approach for studying chronic HBV infection, it is not an appropriate model to assays HBV cccDNA, the HBV infected mice with humanized liver may be an ideal model for further researches. Also, the development of multiplexed CRISPR system delivered *in vivo* by AAV vector would further avoid the HBV escaping from the resistance to single gRNA/Cas9 and enhance the inhibition efficiency (33).

## Availability of data and materials

The Cas9/gRNA dual-expression vector pSpCas9(BB)-2A-Puro (PX459) was obtained from Addgene (plasmid #48139) through an MTA.

## Author contributions

HS, SQ, and HaL designed the study. HaL, CS, HoL, SW, and JZ performed all *in vivo* experiments. JX, LY, and PL performed the bioinformatics analysis. LW, LJ, DX, and YS provided reagents and conceptual advice. HaL and CS wrote the manuscript with comments from all authors.

### Conflict of interest statement

The authors declare that the research was conducted in the absence of any commercial or financial relationships that could be construed as a potential conflict of interest.
