# Supplementary material for: Inhibition of HBV Expression in HBV Transgenic Mice Using AAV-Delivered CRISPR-SaCas9
Source: Front Immunol. 2018 Sep 11;9:2080. doi: 10.3389/fimmu.2018.02080 (PMC6141737; doi:10.3389/fimmu.2018.02080)
Supplement: Supplementary file 1 [file Table_1.DOCX]

Supplementary Table 1. Details of 26 different HBV genotypes.

| Genotype | GenBank ID | Length (bp) | Description |
| --- | --- | --- | --- |
| A | AY034878 | 4653 | Hepatitis B virus isolate HBVKL, complete genome; circular VRL 10-JUN-2001 |
|  | AB194951 | 3221 | Hepatitis B virus DNA, complete genome, isolate CMR82; circμlar VRL 17-NOV-2007 |
|  | AY934772 | 3221 | Hepatitis B virus isolate 1908, complete genome; circμlar VRL 21-JΜL-2005 |
| B | AB287321 | 3215 | Hepatitis B virus DNA, complete genome, isolate Greenland-2; circμlar VRL 6-DEC-2007 |
|  | EF473976 | 3215 | Hepatitis B virus isolate Alr049East, complete genome; circμlar VRL 27-MAY-2008 |
|  | AB219429 | 3215 | Hepatitis B virus DNA, complete genome, isolate Patient #4101; circμlar VRL 06-OCT-2006 |
|  | AB100695 | 3215 | Hepatitis B virus DNA, complete genome, isolate HBV-VH133; circμlar VRL 03-MAY-2003 |
|  | AB010289 | 3215 | Hepatitis B virus DNA, complete genome, isolate Yamagata-1; circμlar VRL 18-DEC-2008 |
|  | AF121245 | 3215 | Hepatitis B virus isolate HBV/16091, complete genome; circμlar VRL 27-FEB-2001 |
| C | AB048705 | 3194 | Hepatitis B virus DNA, complete genome, serotype ayw; circμlar VRL 23-MAR-2001 |
|  | X75665 | 3215 | Human hepatitis virus (genotype C, HMA) preS1, preS2, S, C, X; circμlar VRL 14-NOV-2006 |
|  | AB493837 | 3215 | Hepatitis B virus DNA, complete genome, isolate 03UC; circμlar VRL 04-JUN-2009 |
|  | EU410079 | 3215 | Hepatitis B virus isolate ph109, complete genome; circμlar VRL 11-MAY-2009 |
|  | DQ089778 | 3215 | Hepatitis B virus isolate HK1442, complete genome; circμlar VRL 13-JΜL-2005 |
|  | AB246344 | 3215 | Hepatitis B virus DNA, complete genome, clone C_JPN22; circμlar VRL 28-OCT-2006 |
| D | AB048702 | 3182 | Hepatitis B virus DNA, complete genome, serotype ayw; circμlar VRL 23-MAR-2001 |
|  | EU594435 | 3182 | Hepatitis B virus strain 320-95, complete genome; circμlar VRL 05-AUG-2008 |
|  | AB210822 | 3182 | Hepatitis B virus DNA, complete genome, strain Kokusai-Iryo 2-OS; circμlar VRL 13-MAR-2007 |
|  | AB246348 | 3182 | Hepatitis B virus DNA, complete genome, clone D_US68; circμlar VRL 28-OCT-2006 |
| E | AM494708 | 3212 | Hepatitis B virus complete genome, isolate CAR138; circμlar VRL 08-JAN-2008 |
| F | AB036913 | 3215 | Hepatitis B virus (genotype F) genomic DNA, complete genome; circμlar VRL 20-FEB-2001 |
|  | AY311369 | 3215 | Hepatitis B virus isolate BD4139, complete genome; circμlar VRL 28-AUG-2006 |
|  | AY179735 | 3215 | Hepatitis B virus genotype F isolate BA10, complete genome; circμlar VRL 03-SEP-2003 |
|  | AF223965 | 3215 | Hepatitis B virus strain C-1858 isolate sa16, complete genome; circμlar VRL 17-JAN-2001 |
| G | AB056515 | 3248 | Hepatitis B virus DNA, complete genome, clone USG16; circμlar VRL 04-JUN-2002 |
| H | AY090457 | 3215 | Hepatitis B virus strain 2928Nic, complete genome; circμlar VRL 07-AUG-2002 |

Supplementary Table 2. Sequences of 21 gRNAs that could target the distinct HBV genomes used in this study.

| Name | Sequence (5ʹ-3ʹ) | Target region | |
| --- | --- | --- | --- |
| gRNA-Sa1 | GGCTGCGAGCAAAACAAGCAACT | Polymerase |  |
| gRNA-Sa2 | GACGTAAACAAAGGACGTCCCGCG | HBV X region | |
| gRNA-Sa3 | AATTCTTTGACATACTTTCCAATC | Polymerase | |
| gRNA-Sa4 | GGCACAGCTTGGAGGCTTGAACA | Precore+core | |
| gRNA-Sa5 | AAGAAGTCAGAAGGCAAAAAC | Precore+core | |
| gRNA-Sa6 | CTAGAAAATTGAGAGAAGTCC | HBV S region | |
| gRNA-Sa7 | GAGATTGAGATCTTCTGCGAC | Precore+core | |
| gRNA-Sa8 | GTCAACAAGAAAAACCCCGCC | HBV S region | |
| gRNA-Sa9 | CAAGGTCGGTCGTTGACATTGCT | HBV X region | |
| gRNA-Sa10 | CGGCAGACGGAGAAGGGGACGA | HBV X region | |
| gRNA-Sa11 | GAAAATTGAGAGAAGTCCACC | HBV S region | |
| gRNA-Sa12 | GTCTAGACTCTGCGGTATTGT | HBV S region | |
| gRNA-Sa13 | CAGTAGCTCCAAATTCTTTATA | Precore+core | |
| gRNA-Sa14 | GGATGCTGGATCTTCCAAATTA | Precore+core | |
| gRNA-Sa15 | GTCCTCTTATGTAAGACCTTGGG | HBV X region | |
| gRNA-Sa16 | AGACAAAAGAAAATTGGTAACAGC | HBV S region | |
| gRNA-Sa17 | GTACAGCAACATGAGGGAAACA | HBV S region | |
| gRNA-Sa18 | TATATAGAATACCAGCCTTCCA | Polymerase | |
| gRNA-Sa19 | GGAAAGGAAGGAGTTTGCCATT | Polymerase | |
| gRNA-Sa20 | CGCCGACGGGACGTAAACAAAGG | HBV X region | |
| gRNA-Sa21 | AAACCCCGCCTGTAACACGAGC | HBV S region | |

Supplementary Table 3. Top 5 potential off-target sites for gRNA-Sa4 in NCBI37/mm9 genome.

| Sequence | Score | Mismatches | UCSC gene & Locus |
| --- | --- | --- | --- |
| AAGCATCCAAGCTGTGCCTGGAG | 5.7 | 2MMs[5:20] | chr8:-25650266(off-1) |
| AATCCTCCAAGCTGTGCATTGGG | 2.6 | 2MMs[3:18] | chr4:-57114085(off-2) |
| AAGACTCCAGTCTGTGCCTTGAG | 1.3 | 3MMs[4:10:11] | chr19:-45540747(off-3) |
| TGCCCTACAAGCTGTGCCTTGAG | 0.9 | 4MMs[1:2:3:7] | chr1:-182223450(off-4) |
| GGGCCTACAGGCTGTGCCTTGGG | 0.9 | 4MMs[1:2:7:10] | chr12:-12322706(off-5) |

Supplementary Table 4. Primers used in the off-target assay for each potential off-target region.

| Primer | Sequence (5ʹ-3ʹ) |
| --- | --- |
| **off-target1F** | CACCCAGTTATTTCTACCTTGACAAGCTTT |
| **off-target1R** | TAGATTGAGCACAGCCACAGGAGCC |
| **off-target2F** | TTCTCCTATCACCACAACTGTTCTC |
| **off-target2R** | GTCTGGTGTTTCATCACAGCAATAG |
| **off-target3F** | ATTGGGAAAAGAAGGTCTCAGA |
| **off-target3R** | TGAACAAATGATTATTAAAGACCTC |
| **off-target4F** | TGAACAAATGATTATTAAAGACCTC |
| **off-target4R** | CTTCTATGAGGGCTGGTTGGT |
| **off-target5F** | AACCTTGTTTCCCCTGACGGCTCT |
| **off-target5R** | TAGCCCCGGCTGACCTCAAACTT |


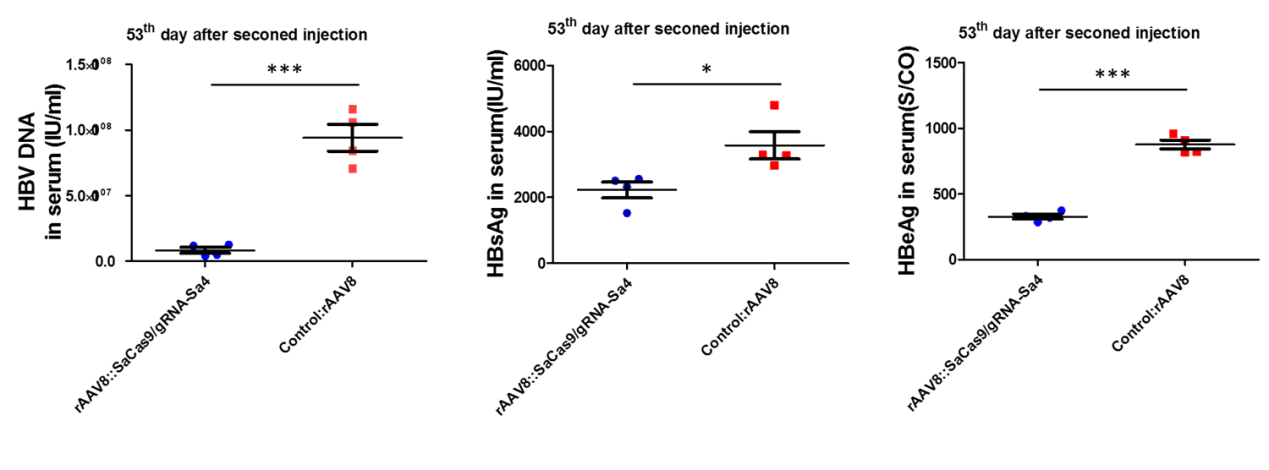


Supplementary Figure 1. Comparison of serum HBV DNA, HBsAg and HBeAg levels measured in rAAV8-empty- and rAAV8::Sa4-treated groups at the 53^th^ day after injection.


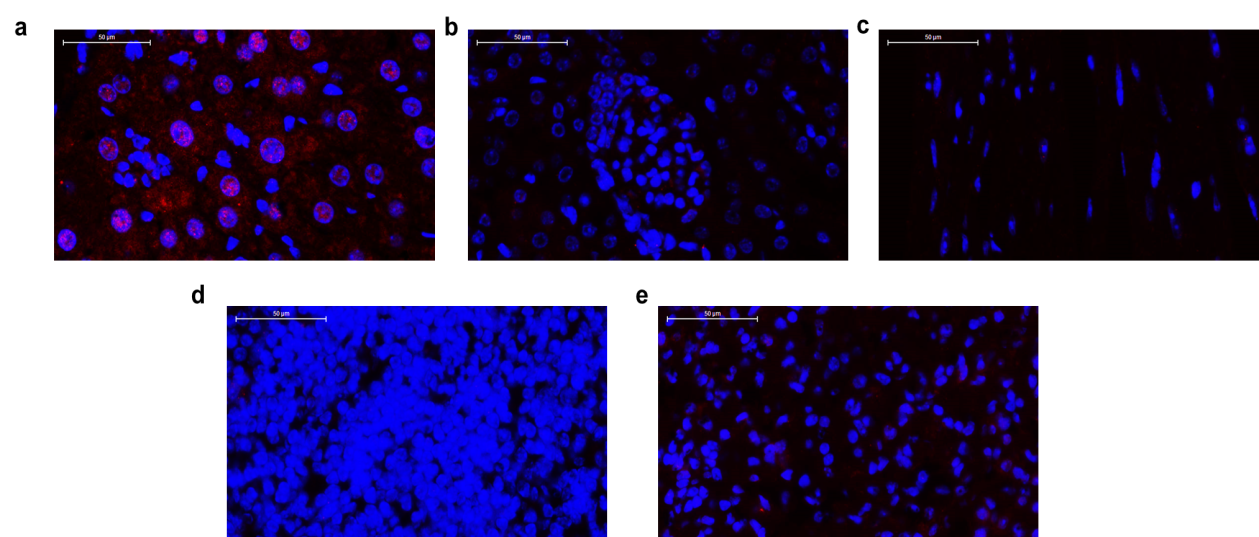


Supplementary Figure 2. Immunofluorescence labeling with anti-HA antibodies showing the levels of AAV::SaCas9-HA (Cy3; red) in the liver (a), kidney (b), heart (c), spleen (d) and lung (e). Nuclei are stained blue with DAPI.


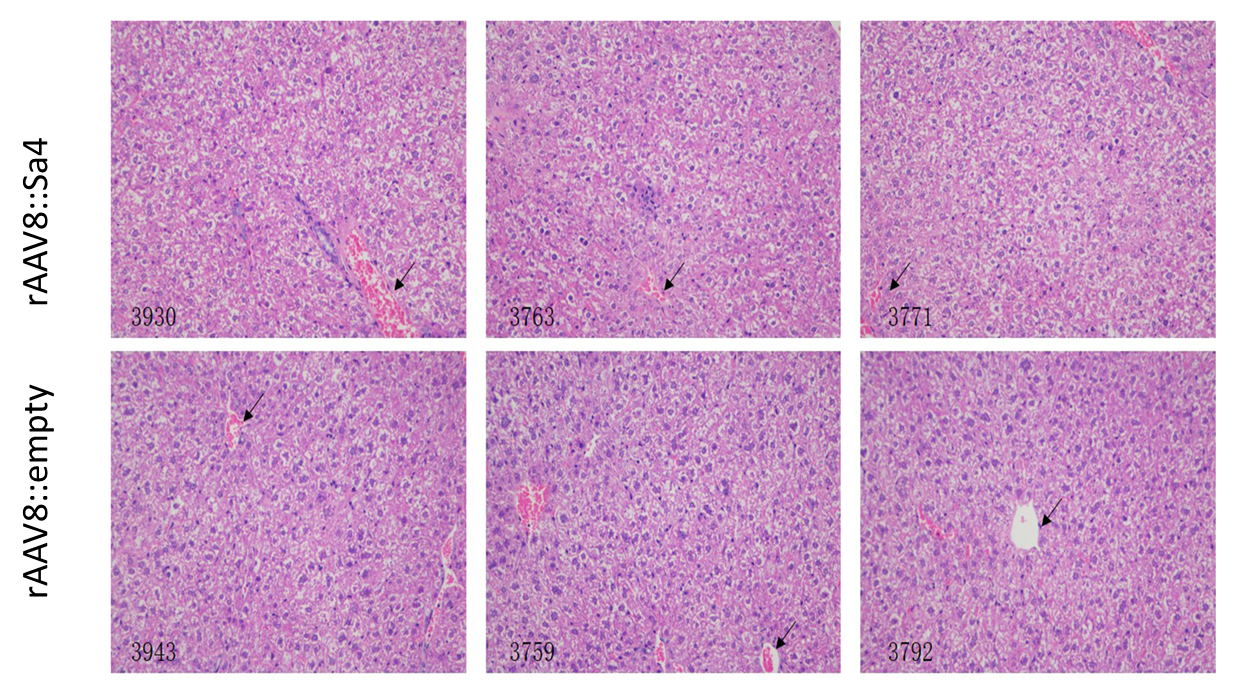


Supplementary Figure 3 Histopathological analysis (HE staining) of liver sections (HBV-Tg mice). The mice in the control and experimental groups presented a normal hepatic cell structure. Central veins are indicated by black arrows (200×).


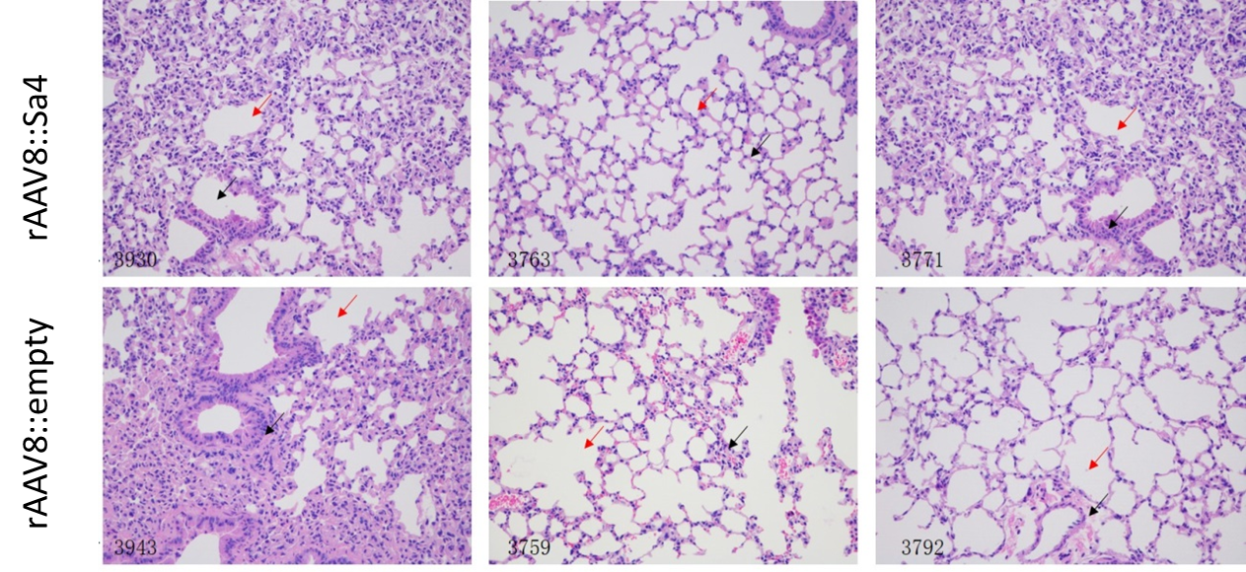


Supplementary Figure 4. Histopathological analysis (HE staining) of lung sections (HBV-Tg mice). The mice in the control and experimental groups showed a normal lung cell structure. Alveoli and bronchi are indicated by red and black arrows, respectively (200×).


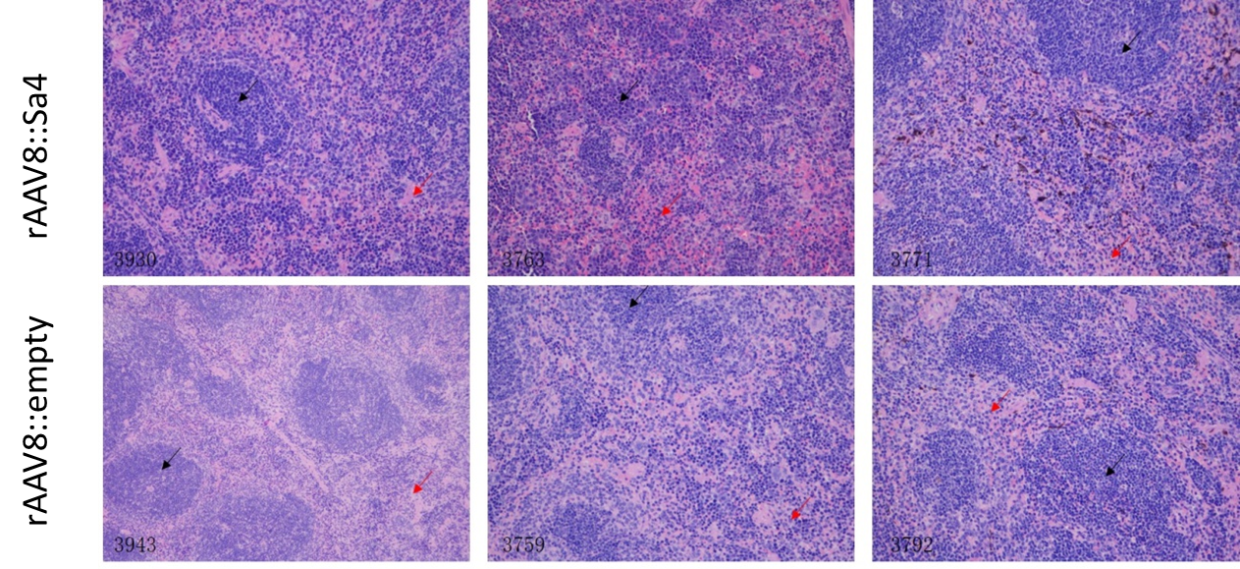


Supplementary Figure 5. Histopathological analysis (HE staining) of spleen sections (HBV-Tg mice). The mice in the control and experimental groups presented a normal spleen cell structure. Splenic white pulp and red pulp are indicated by red and black arrows, respectively (200×).


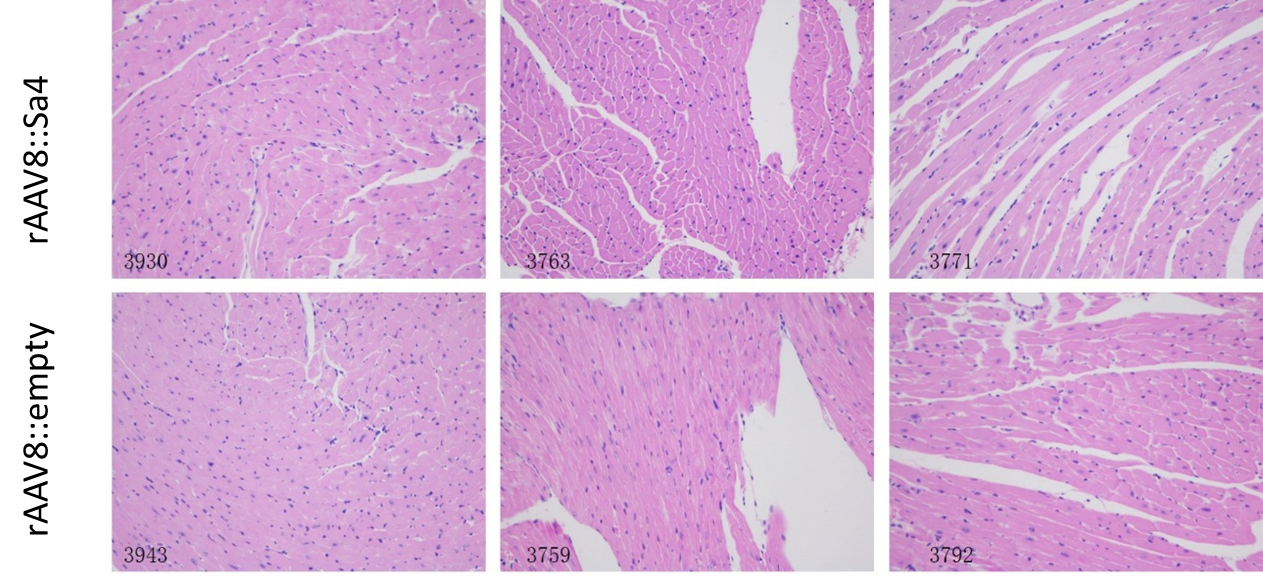


Supplementary Figure 6. Histopathological analysis (HE staining) of heart sections (HBV-Tg mice). The mice in the control and experimental groups showed a normal cardiac cell structure (200×).


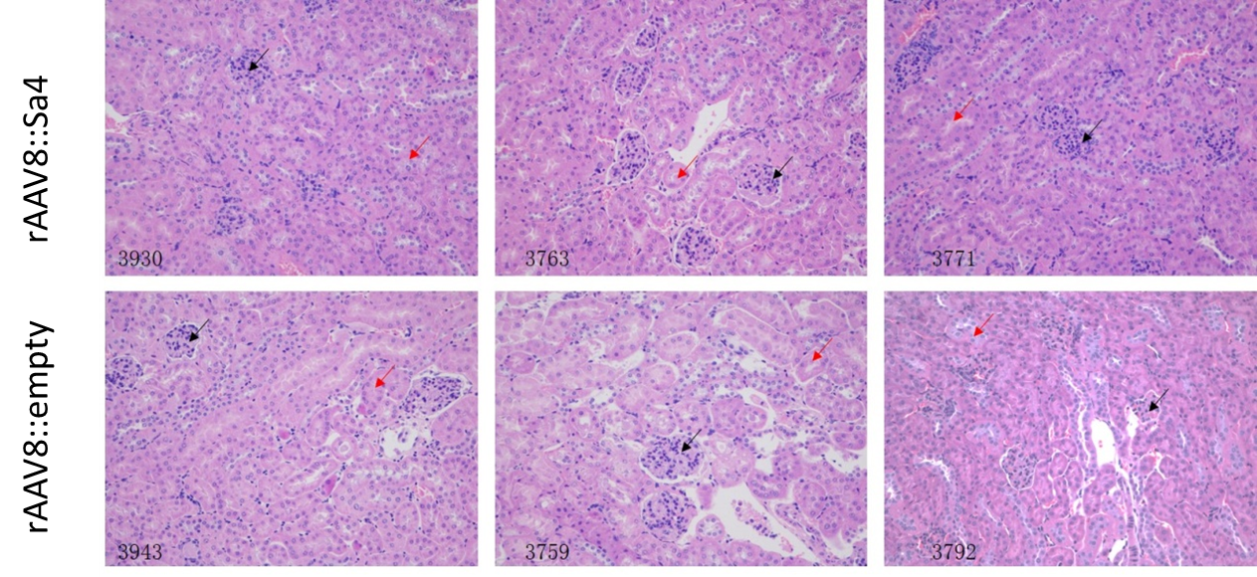


Supplementary Figure 7. Histopathological analysis (HE staining) of kidney sections (HBV-Tg mice). The mice in the control and experimental groups presented a normal renal cell structure. Kidney tubules and glomeruli are indicated by red and black arrows, respectively (200×).
